# Supplementary material for: Microparticles as BDMDAC (Quaternary Ammonium Compound) Carriers for Water Disinfection: A Layer-by-Layer Approach without Biocide Release
Source: Nanomaterials (Basel). 2023 Dec 2;13(23):3067. doi: 10.3390/nano13233067 (PMC10707778; doi:10.3390/nano13233067)
Supplement: Supplementary file 1 [file nanomaterials-13-03067-s001.zip › nanomaterials-2711898-supplementary.pdf]

Supplementary Information

Marta Redondo[1,2], Ana Pereira[1,2], Carlos M Pereira[3], Luís F. Melo[1,2]

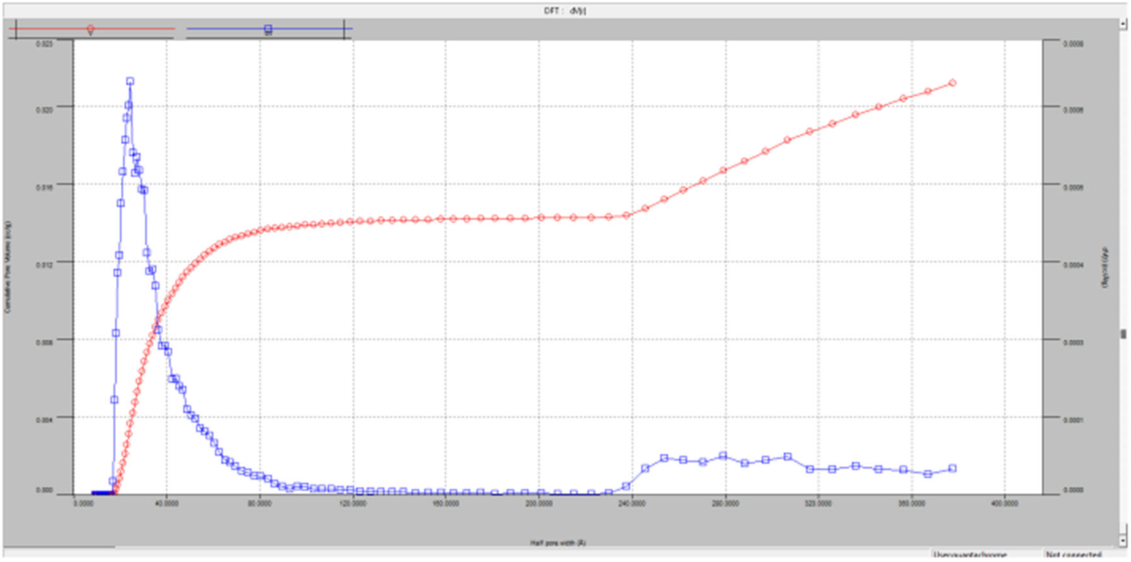

Figure S1. Distribution of pore sizes of $CaCO_3$-LbL.

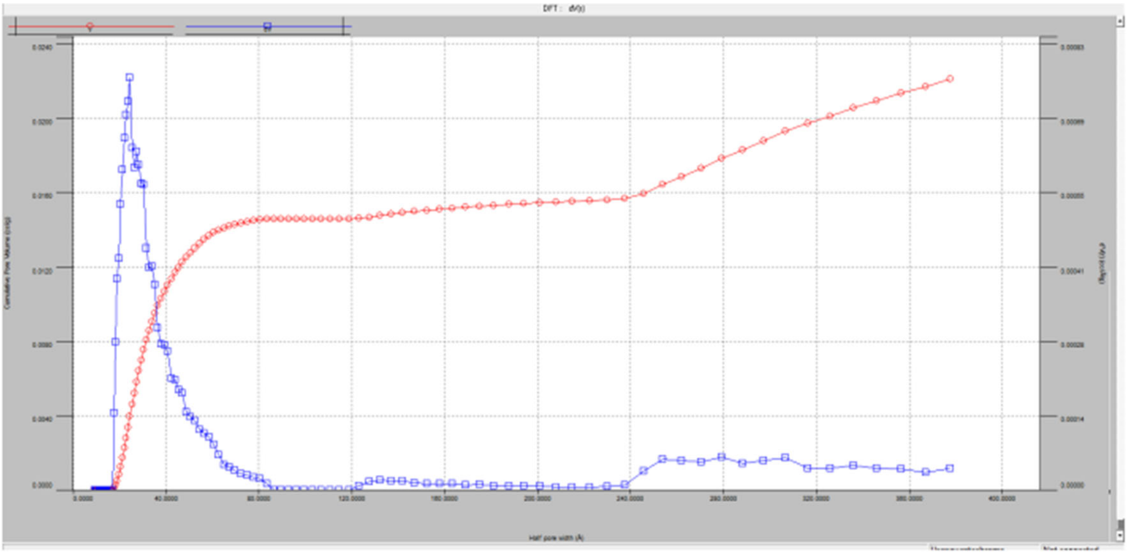

Figure S2. Distribution of pore sizes of $CaCO_3$.

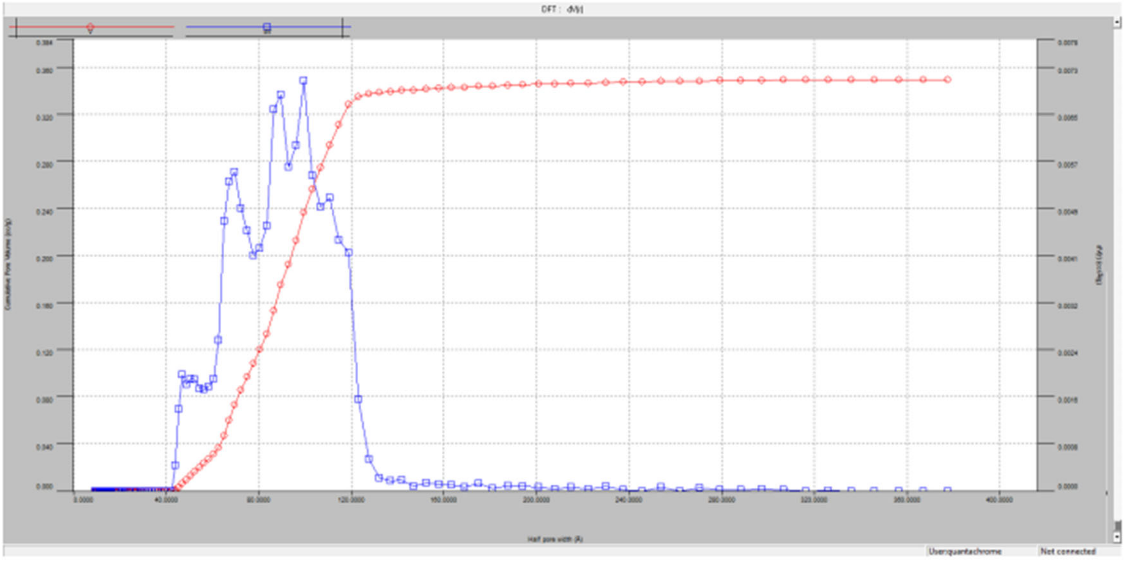

Figure S3. Distribution of pore sizes of Hap-LbL.

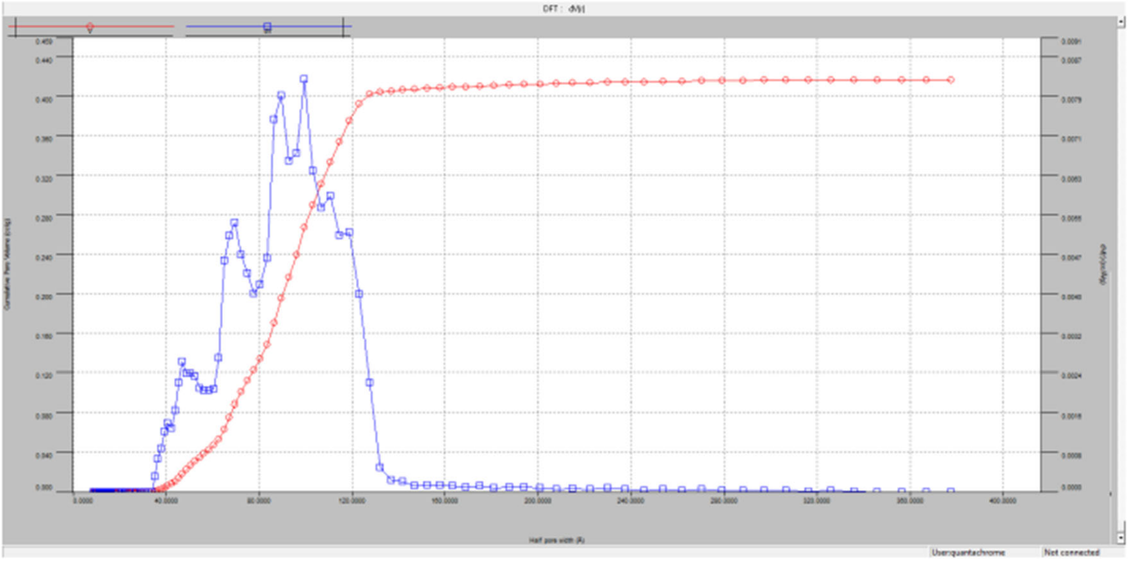

Figure S4. Distribution of pore sizes of Hap.
